# RETRACTION: The Effect of Neoadjuvant Chemotherapy on Surgical Site Wound Infection after Immediate Breast Reconstruction in Patients With Breast Cancer: A Meta‐Analysis

**DOI:** 10.1111/iwj.70314

**Published:** 2025-03-06

## Abstract

**RETRACTION:** ZhangC. , LiJ. , WangL. , SunS. , and ChenC. , “The Effect of Neoadjuvant Chemotherapy on Surgical Site Wound Infection after Immediate Breast Reconstruction in Patients With Breast Cancer: A Meta‐Analysis,” International Wound Journal 21, no. 1 (2024): e14337, 10.1111/iwj.14337.37548134 PMC10777744

The above article, published online on 07 August 2023, in Wiley Online Library (http://onlinelibrary.wiley.com/), has been retracted by agreement between the journal Editor in Chief, Professor Keith Harding; and John Wiley & Sons Ltd. Following an investigation by the publisher, all parties have concluded that this article was accepted solely on the basis of a compromised peer review process. The editors have therefore decided to retract the article. The authors did not respond to our notice regarding the retraction.

**RETRACTION:** C. Zhang , J. Li , L. Wang , S. Sun , and C. Chen , “The Effect of Neoadjuvant Chemotherapy on Surgical Site Wound Infection after Immediate Breast Reconstruction in Patients With Breast Cancer: A Meta‐Analysis,” International Wound Journal 21, no. 1 (2024): e14337, 10.1111/iwj.14337.37548134 PMC10777744

The above article, published online on 07 August 2023, in Wiley Online Library (http://onlinelibrary.wiley.com/), has been retracted by agreement between the journal Editor in Chief, Professor Keith Harding; and John Wiley & Sons Ltd. Following an investigation by the publisher, all parties have concluded that this article was accepted solely on the basis of a compromised peer review process. The editors have therefore decided to retract the article. The authors did not respond to our notice regarding the retraction.

